# Analysis of Cumulative Antibiogram Reports in Search for Optimal Empirical Urinary Tract Infection Treatment at the Central Teaching Hospital of the Medical University of Lodz, Poland: Results of a 3-Year Surveillance

**DOI:** 10.3390/jcm12196270

**Published:** 2023-09-28

**Authors:** Filip Bielec, Monika Wenecka, Małgorzata Brauncajs, Dorota Pastuszak-Lewandoska

**Affiliations:** 1 Department of Microbiology and Laboratory Medical Immunology, Medical University of Lodz, 90-151 Lodz, Poland; monika.wenecka@umed.lodz.pl (M.W.); malgorzata.brauncajs@umed.lodz.pl (M.B.); dorota.pastuszak-lewandoska@umed.lodz.pl (D.P.-L.); 2 Medical Microbiology Laboratory, Central Teaching Hospital of Medical University of Lodz, 92-213 Lodz, Poland

**Keywords:** urinary tract infection, cumulative antibiogram, antimicrobial stewardship, infection prevention and control, antimicrobial resistance, empirical therapy

## Abstract

Urinary tract infections are among the most common bacterial infections, accounting for about two-fifths of all healthcare-associated infections. Appropriate antimicrobial therapy is crucial, e.g., to avoid prolonged hospitalization and limit antimicrobial resistance spread. This study was performed to analyze the microbiological profiles of urinary tract infections in the Central Teaching Hospital in Lodz, Poland, and develop local empirical therapy guidelines. This study was a 3-year retrospective surveillance of the cumulative antibiograms from urine cultures. The procedures were based on the current EUCAST and CLSI guidelines. In 2020–2022, a total of 4656 urine cultures were performed, of which 1134 were positive. The most common bacterial isolates were *Escherichia coli*, followed by *Klebsiella* spp. and *Enterococcus* spp. High levels of susceptibility (>90%) have been observed for carbapenems, piperacillin/tazobactam, amikacin, and nitrofurantoin. Development of the appropriate empirical antimicrobial is a challenging task with persistently high levels of resistance to commonly used antimicrobials. Eventually, we separated the uncomplicated and complicated urinary tract infections in local guidelines and recommended nitrofurantoin and amikacin, respectively, in empiric therapy. The clinicians should make a decision based on the presented symptoms and then—with the urine culture result—correct or continue the therapy.

## 1. Introduction

Urinary tract infection (UTI) is a disease caused by an infection of any part of the urinary tract. UTI is most often the result of the entry of pathogens by the ascending route through the urethra, less often by the descending route (hematogenous or lymphatic) [1,2]. It should be remembered that the human urinary tract is not physiologically sterile, so not every presence of microorganisms in the microbiological urine culture will be indicative of UTI [3]. UTIs are among the most common bacterial infections worldwide, accounting for about 40% of all healthcare-associated infections (HA-UTI) and about 20% of community-acquired infections (CA-UTI). It is estimated that about half of women and one-tenth of men experience an episode of UTI in their lifetime [2,4].

Gram-negative bacilli are the most common etiologic factors of UTI [1]. In addition to bacterial agents, fungi (mainly *Candida* spp. And, less frequently, others) can also be the etiology of UTI. The meaning of *Candida* spp. in the pathogenesis of various diseases is increasing, especially in critically ill patients [5]. Correct diagnosis of fungal UTI may be a big challenge for clinicians because the presence of *Candida* spp. in the urine culture may represent sample contamination, physiological colonization, or, eventually, infection. Contamination can often be differentiated by obtaining a new urine sample for microbiological examination and checking whether the *Candida* spp. finding persists. Unfortunately, this practice is uncommon, which may result in the unjustified use of antifungals [3,6].

Current UTI treatment guidelines classify the condition into uncomplicated and complicated UTIs. Uncomplicated UTI is defined as “acute, sporadic or recurrent lower (uncomplicated cystitis) and/or upper (uncomplicated pyelonephritis) UTI, limited to nonpregnant women with no known relevant anatomical and functional abnormalities within the urinary tract or comorbidities”. On the other hand, complicated UTI is defined as “all UTIs which are not defined as uncomplicated” [7]. In complicated UTIs, the tissues inside are mainly infected, not just the urine and the epithelium in contact with it, as in uncomplicated UTIs. This affects the selection of recommended antimicrobials, which differ in their pharmacological properties [2,7].

Bacterial antimicrobial resistance poses a significant challenge to the effective clinical management of infections, including UTIs. The development of resistant microorganisms can happen naturally, regardless of antimicrobial use. Nevertheless, there is evidence indicating that the misuse and overuse of antimicrobials remain the primary causes of antimicrobial resistance, providing the necessary selective pressure for the emergence and spread of resistant strains. In healthcare settings, the propagation of antimicrobial-resistant organisms limits available treatment options, resulting in prolonged hospital stays, increased expenses, poor prognosis, and higher mortality rates. These factors emphasize the need to preserve existing antimicrobials for improved patient outcomes and health system benefits [8,9].

One of the antimicrobial stewardship interventions that may improve prescribing practices is the development of local hospital empiric therapy guidelines. This can be achieved by constructing and analyzing cumulative antibiograms [8]. Development of the optimal empirical therapy guidelines requires an understanding of local epidemiology and antimicrobial susceptibility. One of the European Association of Urology (EAU) recommendations is to monitor local resistance patterns of common uropathogens so that empirical therapy guidelines can be tailored to the local epidemiological situation [7]. Cumulative antibiograms are combined data from microbiological tests in a specified period in a given healthcare unit. They may apply to all locally ordered microbiological tests, selected biological material, or specific groups of patients. They describe the percentage of microorganisms that remained susceptible to chosen antimicrobials. Cumulative antibiograms performed locally in hospitals are the most useful empirical guide for selecting antimicrobial treatments for common infections and may form the basis for local guidelines [10,11].

This study aimed to analyze the microbiological profile of UTIs in the Central Teaching Hospital of the Medical University of Lodz, Poland. The specific objectives included (1) the evaluation of changes in the etiology of the UTIs and the antimicrobial susceptibility of isolated strains between 2020–2022; (2) the analysis of the current state of antimicrobial susceptibility, and on this basis, the development of local empirical treatment guidelines for UTIs.

## 2. Materials and Methods

This study was a 3-year retrospective surveillance of the microbiological reports from positive urine cultures held in the Central Teaching Hospital of the Medical University of Lodz, Poland. The part of the hospital under study treats adult patients and consists of an emergency room, 10 medical wards, 3 surgical wards, 3 intensive care wards, and 6 psychiatric wards. Data from the years 2020–2022 were obtained from the local Medical Microbiology Laboratory—including bacterial identification and antimicrobial susceptibility testing—and from the Infection Prevention & Control Team—concerning hospital-associated UTI. Results indicating the presence of fungi in urine samples were excluded from this study. In only a few cases, the presence of the fungi in urine samples was verified by repeated testing.

Urine samples for microbiological analysis were collected from the patients by either clean catch midstream or diagnostic catheterization, according to indications, the patient’s condition, and local hospital guidelines. Samples were immediately transported to the laboratory and then secured at 2–5 °C for 20 h maximum before culturing. The urine was quantitatively streaked on an agar media—Columbia w/5% sheep blood, MacConkey, and Coccosel (Thermo Fisher Scientific, Waltham, MA, USA)—with calibrated 1 µL loop and then incubated for 18 ± 2 h at 35 ± 1 °C in atmospheric conditions. Samples were considered positive with the growth of one microbial species in the count ≥10^5^ CFU/mL from midstream and ≥10^3^ CFU/mL from a catheter or for other reasons (more species, different counts) if the patient’s physician indicated so after telephone consultation. Bacterial species were identified using matrix-assisted laser desorption/ionization-time of flight mass spectrometry (MALDI-TOF MS) technology with the VITEK MS system (bioMérieux, Marcy-l’Étoile, France). Antimicrobial susceptibility testing was carried out automatically with the BD Phoenix system (Becton, Dickinson and Company, Franklin Lakes, NJ, USA) from January 2020 to June 2021 and the VITEK2 system (bioMérieux, Marcy-l’Étoile, France) from July 2021 to December 2022. Susceptibility for fosfomycin and nitroxoline was determined by disc diffusion according to the European Committee on Antimicrobial Susceptibility Testing (EUCAST) methodology [12]. Clinical interpretation of antimicrobial susceptibility was determined according to the current EUCAST guidelines [13]—for nitrofurantoin, *Enterococcus faecalis* breakpoints were used for the entire genus of *Enterococcus*. Epidemiologically and clinically important resistance mechanisms were detected following appropriate EUCAST guidelines [14].

Nosocomial infections were identified by the Infection Prevention & Control Team based on continuous analysis of current microbiological test results, patients’ medical records, and clinicians’ reports. The definitions of HA-UTI were taken from the guidelines of the European Center for Disease Prevention and Control (ECDC) [15], based on which local hospital procedures have been developed.

Cumulative antibiograms were prepared according to Clinical and Laboratory Standards Institute (CLSI) guidelines [10]. We included in the analysis all positive bacteriological urine cultures based on the methodology used in the hospital’s Medical Microbiology Laboratory, as described above. Only the first isolate per patient with verified final identification and susceptibility testing results was included. The first isolate refers to the initial microbial isolate of a particular species recovered from a patient’s urine during the analyzed periods–separately for each year 2020, 2021, and 2022. Susceptible isolates include all strains interpreted by EUCAST as “susceptible” (S) or “susceptible, increased exposure” (I) [13].

All data were descriptively analyzed using MS Excel 2021 (Microsoft, Redmond, WA, USA). Statistical analysis was performed using Statistica 13 (TIBCO, Palo Alto, CA, USA). The distribution of collected data was checked using the Shapiro–Wilk test. The chi-square test and the sign test were used to compare the differences. A *p*-value of 0.05 was considered the limit of statistical significance. Before inclusion in the analysis, data accuracy, completeness, and clarity were checked.

## 3. Results

In the entire period of 2020–2022, a total of 4656 microbiological urine cultures were performed, of which 1134 (24.36%) turned out to be positive for the presence of a bacterial pathogen. Only the first isolates in each study year were considered for further analysis, following the CLSI guidelines [10]–to avoid falsely overestimating antibiotic resistance levels caused by repeated testing of samples from the same patients (usually difficult to treat) [16].

**Figure 1:**
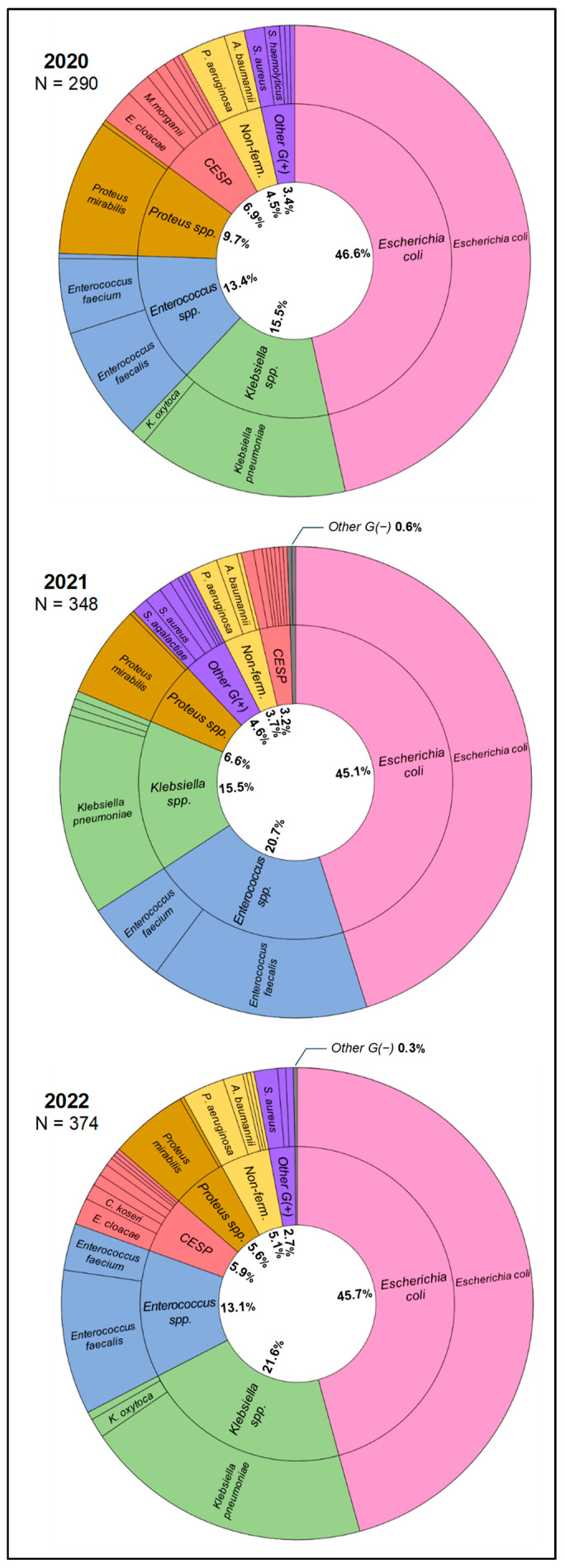
Urinary tract infection etiological factors in the studied period. Abbreviations: CESP—group of *Citrobacter* spp., *Enterobacter* spp., *Serratia* spp., *Providencia* spp., *Morganella* spp., and *Hafnia* spp.; Non-ferm.—group of non-fermenting Gram-negative bacilli.

Figure 1 presents the bacteria identified in urine samples in the studied period. Microorganisms were grouped according to microbiological similarity—*Escherichia coli*, *Klebsiella* spp., *Enterococcus* spp., *Proteus* spp., non-fermenters, CESP group, and others. According to Gram staining, Gram-negative bacteria accounted for 83%, 79%, and 84% of the UTI etiological factors in 2020, 2021, and 2022, respectively, and Gram-positive bacteria—17%, 21%, and 16%, respectively. Statistical tests showed no significant differences in the UTI etiology between the studied years–both when comparing all identified species and the groups.

In each studied year, most positive urine cultures came from women—69.7%, 74.1%, and 70.9% in 2020, 2021, and 2022, respectively. When comparing the identified pathogens by gender, statistically significant differences were observed for the most common etiological factors of UTI. *E. coli* was always the most frequently identified pathogen, but while in the case of women, the frequency was more than half (50.6–55.9%), in the case of men, it ranged from 25.0 to 33.9%. On the other hand, *Enterococcus* spp. was identified much more frequently in men than in women (19.3–23.9% vs. 8.9–19.8%, respectively), which was the second most common cause of UTI in men before *Klebsiella* spp. in each studied year. Comparing the collected data by patients’ age (<65 years old vs. 65+), no statistically significant differences were observed.

**Figure 2:**
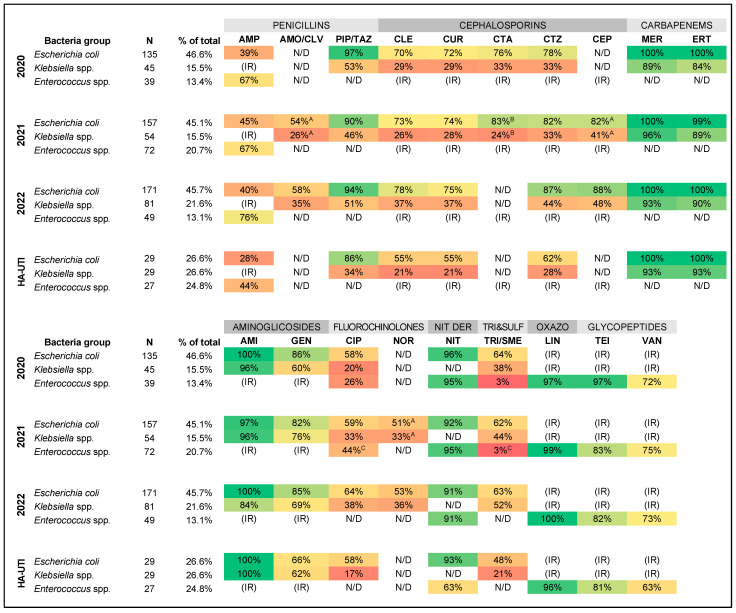
Cumulative antibiograms from urine samples in the studied period. The color of the cells correlates with the level of antimicrobial susceptibility—the greener, the more susceptible; the redder, the more resistant. Due to the change in automated systems used in the microbiology laboratory in the half of 2021, not all isolates from this year were tested with some antimicrobials: ^A^ 76 *Escherichia coli* and 25 *Klebsiella* spp. tested from the beginning of July; ^B^ 81 *E. coli* and 29 *Klebsiella* spp. tested until the end of June; ^C^ 36 *Enterococcus* spp. tested until the end of June. Healthcare-associated infections (HA-UTI) data are aggregated for the entire 2020–2022 period and are indicative only, as no bacteria group reached a statistical significance. Abbreviations: NIT DER—nitrofuran derivatives; TRI&SULF—trimethoprim and sulfonamides; OXAZO—oxazolidinones; AMP—ampicillin; AMO/CLV—amoxicillin/clavulanic acid; PIP/TAZ—piperacillin/tazobactam; CLE—cephalexin; CUR—cefuroxime; CTA—cefotaxime; CTZ—ceftazidime; CEP—cefepime; MER—meropenem; ERT—ertapenem; AMI—amikacin; GEN—gentamicin; CIP—ciprofloxacin; NOR—norfloxacin; NIT—nitrofurantoin; TRI/SME—trimethoprim/sulfamethoxazole; LIN—linezolid; TEI—teicoplanin; VAN—vancomycin; N/D—no data; (IR)—intrinsic resistance).

Cumulative antibiograms for the studied period were developed only for groups (species/genera) in which the number of unique first isolates in a given period was ≥30. This condition was met only by *E. coli*, *Klebsiella* spp., and *Enterococcus* spp.—in each studied year, these bacteria accounted for over 75% of UTIs (see Figure 1). Cumulative antibiograms are presented in Figure 2. Statistical tests showed no significant differences in cumulative antibiograms between the years studied. Among Gram-negative bacilli, resistance to the tested antibiotics was significantly higher in *Klebsiella* spp. than in *E. coli* in each year.

Drug susceptibility to fosfomycin and nitroxoline has been determined for only 33 *E. coli* strains from the end of 2022. They showed 100% susceptibility to fosfomycin and 97% susceptibility to nitroxoline.

**Figure 3:**
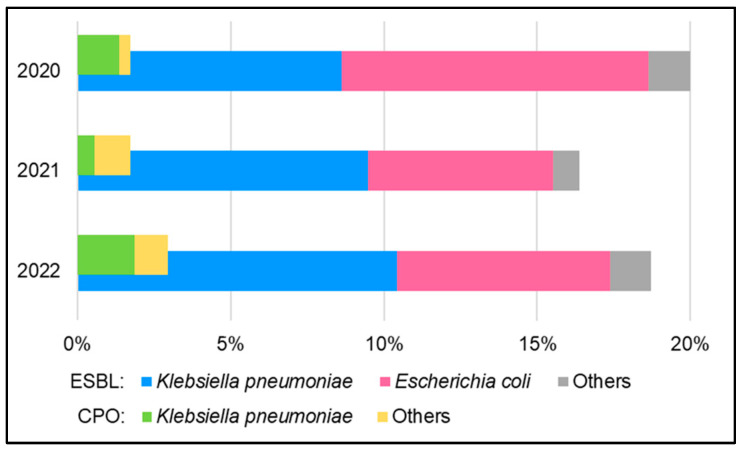
The frequency of ESBL producers and CPO identified in urine samples in the studied period.

The frequency of extended-spectrum β-lactamases (ESBL) producers and carbapenemase-producing organisms (CPO) among the identified Gram-negative bacilli was also calculated—the results are presented in Figure 3. Statistical tests showed no significant differences in the frequency of ESBL producers and CPO between the studied years (however, it should be noted that due to the low number of CPOs in general, the power of the tests was low).

### Healthcare-Associated Infections

**Figure 4:**
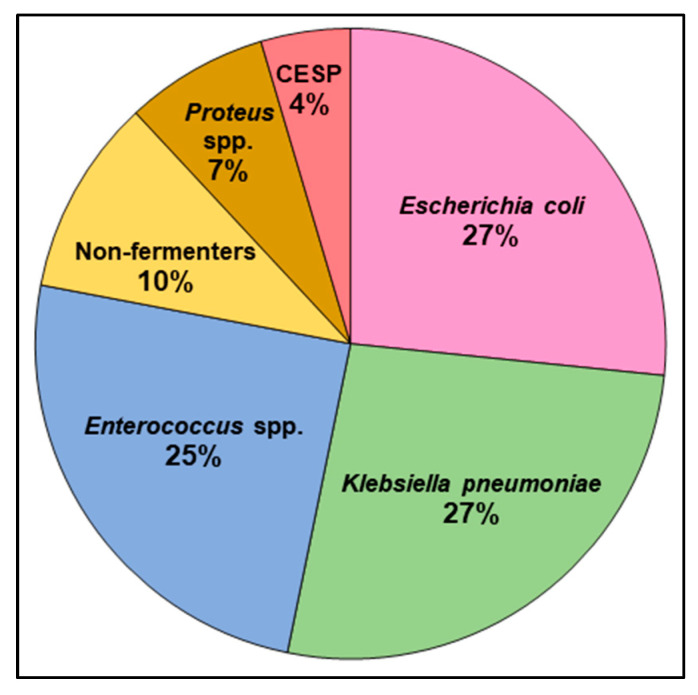
Healthcare-associated urinary tract infection etiological factors in the studied period cumulatively. Abbreviation: CESP—group of *Citrobacter* spp., *Enterobacter* spp., *Serratia* spp., *Providencia* spp., *Morganella* spp., and *Hafnia* spp.

Throughout 2020–2022, a total of 103 bacterial HA-UTIs were identified, representing 9.08% of all microbiologically confirmed cases of UTI. Figure 4 presents the distribution of HA-UTI etiological factors. Due to the generally low frequency of HA-UTI in the observed hospital, even when cumulating all cases from 3 years, it was impossible to reach a statistically significant group for the analysis of cumulative antibiograms. However, Figure 2 presents the indicative cumulated data for informational purposes.

## 4. Discussion

### 4.1. Etiological Factors

The most common UTI etiological factor was *E. coli*, accounting for approximately 45% of all identified urinary pathogens in the years studied. However, the percentage of *E. coli* observed in our 3-year analysis was significantly lower than the generally accepted range of 65–75%, replicated by a 2015 publication in Nature Reviews Microbiology [1]. A more accurate summary of the general UTI epidemiology seems to be our review based on data from the last 10 years, published previously [17]. The decrease in the importance of *E. coli* in the UTI etiology may be due to the good management of these infections in outpatient care, suggesting that uropathogenic *E. coli* remains susceptible to commonly used antibiotics. Such a situation could be considered positive. On the other hand, eradicating susceptible bacteria with excessive antibiotic therapy may cause an increase in the frequency of UTIs caused by more resistant pathogens.

Apart from *E. coli* and *Klebsiella* spp., a high percentage (exceeding 10% each year) of UTI etiological factors was *Enterococcus* spp., which is in concordance with other published data [1,18,19,20]. It should be noted that bacteria of the genus *Enterococcus* in low titers may constitute a natural microbiota of the urinary tract (urobiota) [3]. In addition, some hospitals use boronic acid to preserve urine samples for microbiological cultures—Meers et al. [21] observed that *Enterococcus* spp. is resistant to the inhibitory effects of this substance. However, this type of transport medium with a preservative is not used in our hospital. It is recognized that UTI pathogens often migrate from other ecological niches of the body—intestinal or vaginal microbiota [22]. Antimicrobial interventions (local or systemic) targeting these regions may influence the epidemiology of UTIs. Excessive use of, e.g., trimethoprim/sulfamethoxazole or fluoroquinolones in therapy (both in outpatients and inpatients) leads to the eradication of susceptible microorganisms, expanding the colonization possibilities of bacteria resistant to these drugs, such as *Enterococcus* spp.

Considering only isolates classified as HA-UTI, the epidemiology was slightly different. Gram-negative bacilli were in the majority again, but the proportion of *E. coli* decreased in favor of *K. pneumoniae*. Among the most common pathogens causing HA-UTI was also the *Enterococcus* spp. An important observation in comparing healthcare-associated infections with the general etiology of UTI is the twice-as-high share of Gram-negative nonfermenting bacilli, characterized by a broad spectrum of antimicrobial resistance. The observed results are consistent with the HA-UTI overview based on publications from the last 10 years published previously [17] and the results of the ECDC 2016–2017 point prevalence survey [23].

### 4.2. Antimicrobial Susceptibility

Of the penicillins analyzed, only piperacillin/tazobactam remained in >90% active to *E. coli*. For *Klebsiella* spp., piperacillin/tazobactam susceptibility remained only around 50%. For the remaining bacteria–antibiotic pairs, none showed appropriate susceptibility to penicillins to be recommended for empiric therapy. Susceptibility to penicillins remained at a similar level during the studied period. A slight increase in susceptibility to ampicillin in *Enterococcus* spp. resulted from an increase in the ratio of identified *E. faecalis* to *Enterococcus faecium* (the latter is intrinsically resistant to ampicillin).

None of the tested cephalosporins showed >90% activity against the analyzed bacteria. Susceptibility >80% was found in *E. coli* but only in 3rd and 4th generation cephalosporins. There was a slight tendency of increased susceptibility to cephalosporins among *E. coli* and *Klebsiella* spp. *Enterococcus* spp. are intrinsically resistant to cephalosporins [24], which ultimately excludes using those antibiotics in UTI empirical therapy, whereas *Enterococcus* spp. constitute a significant number of pathogens.

Carbapenems are a group of beta-lactams reserved for the treatment of infections caused by microorganisms resistant to other antibiotics [25]. Currently, they have no place in empirical therapy, and the analysis of susceptibility to carbapenems in cumulative antibiograms serves only epidemiological purposes. There was a high level of susceptibility to meropenem and ertapenem among *E. coli* and *Klebsiella* spp. during the studied period. There were no data for *Enterococcus* spp. as our laboratory does not routinely test Gram-positives for carbapenem susceptibility.

UTIs are the only indication for which EUCAST allows the use of aminoglycosides as monotherapy [13]. During this study period, gentamicin maintained a similar level of activity against the analyzed Gram-negative bacilli. For *E. coli*, it was >80%, but for *Klebsiella* spp. <80%, which disqualifies this antibiotic from empirical therapy. In the case of amikacin, persistently high susceptibility of *E. coli* and *Klebsiella* spp. >80% was observed in the studied period (for *E. coli* alone, it was >90%, and in the two studied years, even 100%). *Enterococcus* spp. shows intrinsically low-level resistance to aminoglycosides. However, the combination of aminoglycosides with cell wall synthesis inhibitors (beta-lactams or glycopeptides) may be synergistic [24]. Therefore, amikacin remains in the spectrum of potential antibiotics recommended in empirical treatment—we discussed this later.

All analyzed bacteria—*E. coli*, *Klebsiella* spp., and *Enterococcus* spp.–showed a high level of resistance to ciprofloxacin and norfloxacin. Analyzing the trends of changes in resistance, a slight decrease in resistance to fluoroquinolones could be observed in the studied period. This is probably because of the decreasing use of these antibiotics due to high levels of resistance to fluoroquinolones in Europe [26]. These drugs should also be limited to targeted therapy of susceptible microbes due to the serious adverse effects [27].

Both *E. coli* and *Enterococcus* spp. showed high susceptibility >90% to nitrofurantoin. The laboratory did not report *Klebsiella* spp. susceptibility to this antibiotic because EUCAST did not specify breakpoints for this type of bacteria [13]. *Klebsiella* spp. is in the spectrum of action of nitrofuran derivatives. Therefore, the lack of susceptibility data does not preclude their recommendation for empiric therapy. Nitrofurantoin does not reach therapeutic concentrations in tissues but only in urine [28], so it should only be used in uncomplicated UTIs [7,20]. A characteristic problem for Poland is the unavailability of nitrofurantoin on the market. Its analog, furazidin, is used instead. However, nitrofurantoin susceptibility can be reasonably extrapolated to furazidin, as we have demonstrated experimentally [17]. In the analysis of cumulative antibiograms from our hospital, a slight trend of susceptibility decrease to nitrofuran derivatives was observed. It might be caused by furazidin overuse, which is available over the counter in Poland. In addition, many medical professionals do not see this substance as a standard antimicrobial.

Low levels of susceptibility to trimethoprim/sulfamethoxazole have been observed in *E. coli*, *Klebsiella* spp., and *Enterococcus* spp. Our laboratory does not test susceptibility for trimethoprim alone, but those data would be without meaning—the resistance would be even higher. The relevance of trimethoprim alone susceptibility testing would be justified in the case of low trimethoprim/sulfamethoxazole resistance, while we would like to protect this antimicrobial combination.

During the studied period, *Enterococcus* spp. showed >90% susceptibility to linezolid, >80% to teicoplanin, and >70% to vancomycin. All Gram-negatives are intrinsically resistant to oxazolidinones (linezolid here) and glycopeptides (teicoplanin and vancomycin here) [24]. Therefore, they should not be used in the empirical therapy of UTIs, which are mainly caused by Gram-negative bacilli. In addition, antibiotics from these groups should be reserved for the targeted therapy of infections by Gram-positive cocci resistant to other antibiotics [25].

The analysis of cumulative antibiograms for oxazolidinones and glycopeptides is primarily for epidemiological purposes. The high percentage of vancomycin-resistant enterococci (VRE) strains appeared alarming and needed to be reviewed and addressed by the hospital’s Infection Prevention & Control Team. The analysis of the resistance mechanisms frequency among Gram-negative bacilli (ESBL and CPO) is of similar importance.

Other interesting candidates for UTI empirical therapy are drugs not routinely tested by the hospital microbiology laboratory in the studied period—fosfomycin (oral) and nitroxoline. Antimicrobial susceptibility testing of all urinary *E. coli* for these antibiotics was started in our unit at the end of 2022. Therefore, the test results were not included in the general analysis. The high susceptibility (fosfomycin 100%, nitroxoline 97%) indicated the great potential of these antibiotics in empirical therapy—more on this can be discussed once data are available over more extended periods and for more significant numbers of strains. Both fosfomycin and nitroxoline are active against susceptible strains of *Klebsiella* spp. [29,30] and *Enterococus* spp. [30,31]; however, EUCAST [13] has not yet developed breakpoints allowing standardized susceptibility testing. The use of nitroxoline in the UTI empirical treatment is also supported by its antifungal activity [30], which is difficult to assess definitively due to the problem of definitive interpretation of positive mycological cultures from urine.

### 4.3. Development of Local Empirical Treatment Guidelines for UTIs

Choosing the right antimicrobial for the UTI empirical treatment in our hospital was challenging. Few of the tested antimicrobials recommended in the UTI first-line therapy showed activity in >90% of the tested bacteria groups, which accounted for the majority of etiological factors. A reasonable choice could be amikacin, which is approved as monotherapy for UTI [13,20].

Unfortunately, aminoglycosides showed low activity against *Enterococcus* spp., which accounted for >10% of UTI pathogens. The idea of adding the beta-lactam or glycopeptide to amikacin in empirical therapy could be considered—such combinations are synergistic and bactericidal for enterococci, which are not resistant to cell wall synthesis inhibitors and are not high-level resistant to aminoglycosides [24]. In our hospital, we observed a high cumulative resistance of *Enterococcus* spp. to beta-lactams. Glycopeptides should be reserved for the treatment of infections caused by Gram-positive cocci resistant to other antibiotics [25]. An interesting alternative—however, requiring much more research—would be a combination of amikacin and nitrofurantoin (or furazidin). These antibiotics have a proven—in vitro and an animal model—synergistic effect against uropathogenic *E. coli* [32].

The analyzed bacteria groups in our hospital showed a high susceptibility of >90% to both amikacin and nitrofurantoin. There was no information on the cumulative susceptibility to nitrofurantoin for *Klebsiella* spp., but we do know that nitrofuran derivatives are potentially active against these bacteria. The recommendation of nitrofurantoin (furazidin) in the empirical therapy of UTI would be a reasonable solution (high susceptibility documented in about 60% of UTI pathogens). Nitrofuran derivatives’ pharmacokinetic properties [28], which limit their use to uncomplicated UTIs [7,20], are the only arguments against it.

### 4.4. Comparison to Data from Other Hospitals

Recently, similar analyses from other hospitals in Poland have appeared in the literature. Kot et al. [33] presented cumulative results for uropathogens isolated in the first half of 2020 for the district hospital in Wołomin, Central Poland. Michno et al. [34] presented data for the district hospital in Tarnów, Southern Poland, from the nephrology department for 3 years (2013–2015). In both analyses, *E. coli* and *Klebsiella* spp. were the most common bacteria cultured from urine samples. However, regarding *Enterococcus* spp. in the Wołomin hospital, it was the third most common uropathogens’ group (15.5%), while in the Tarnów hospital, the percentage of enterococci was only 2.2%, and other Gram-negative bacilli were more frequently identified.

Susceptibility profiles of Gram-negative bacilli in both studies [33,34] were similar to the results obtained in our hospital—high levels of resistance to penicillins, fluoroquinolones, and trimethoprim/sulfamethoxazole; persistently very high susceptibility to carbapenems; and high susceptibility to aminoglycosides and nitrofuran derivatives. Susceptibility to cephalosporins for *E. coli* in the study performed by Kot et al. [33] for a hospital in Central Poland was 67–68%, similar to our results (also from a hospital in Central Poland). In the study of Michno et al. [34], performed in a hospital in Southern Poland, the susceptibility to cephalosporins for *E. coli* was as high as 80–90%. This may indicate regional differences in drug susceptibility profiles within the country.

*Enterococcus* spp. cumulative antibiogram in the study of Kot et al. [33] showed >80% susceptibility for vancomycin only. However, the authors did not include in their analysis the antimicrobial susceptibility testing for nitrofuran derivatives, which showed high in vitro activity in our study.

Another Polish study was conducted by Wanke-Rytt et al. [35] in a large pediatric hospital in Warsaw, Central Poland, for the period 2020–2022. Similar to our analysis of adult patients, the three most common uropathogens in children were *E. coli* (58.6–70.0%), *Klebsiella* spp. (9.2–13.2%) and *Enterococcus* spp. (3.9–9.8%). The antimicrobial susceptibility profiles of these three groups of bacteria showed significantly higher levels of susceptibility to all antibiotic groups than our study. This is most likely because the pediatric population is colonized more with wild strains of bacteria than adults.

Similar objectives to our analysis were set by the authors from other hospitals in Central Europe. Hrbacek et al. [36] analyzed data from 2011 to 2019 for the tertiary hospital urology department in Czechia and Gajdács et al. [37,38] from 2008 to 2017 for the tertiary hospital in Hungary. Similar to our results, the most common uropathogen in both studies was *E. coli*. In the Czech study [36], *Enterococcus* spp. was in second place, and *Klebsiella* spp. was in third place. In the Hungarian study [37], *Klebsiella* spp. was in second place, and Gram-positive cocci in third place—the authors did not distinguish individual genera/species in this group.

In the study by Hrbacek et al. [36], *E. coli* and *Klebsiella* spp. showed higher susceptibility to amoxicillin/clavulanate and gentamicin; susceptibility levels to other antimicrobials were similar. For *Enterococcus* spp., a significantly higher susceptibility to ampicillin and vancomycin was observed in a Czech hospital. Gajdács et al. [38] analyzed antibiograms only for Gram-negative bacilli. Susceptibility levels of *E. coli* and *Klebsiella* spp. to ciprofloxacin, gentamicin, trimethoprim/sulfamethoxazole, and nitrofurantoin were similar to or higher than in our study. The persistent higher susceptibility of the most common uropathogens to different groups of antimicrobials in Czechia and Hungary may indicate a higher quality of antimicrobial stewardship in these countries.

### 4.5. Strengths and Limitations

In this study, we analyzed data on uropathogens from 3 years in a large teaching hospital in Central Poland. Only a few similar publications are available in the scientific databases—our publication complements the data needed for systematic analysis at the national and international levels. The methods used in our medical microbiology laboratory are modern and consistent with international guidelines, thanks to which we are sure of the reliability of the data included in the analysis.

This study has several limitations, which result primarily from the prelaboratory stages. We cannot be sure whether clinicians ordered urine microbiology testing for all patients with symptoms suggestive of UTI. On the other hand, it is possible that urine microbiological testing was ordered for asymptomatic patients without any significant indications. This could have influenced the percentage of positive results and the distribution of individual bacterial species frequency. At the laboratory level, diagnosticians cannot clearly distinguish colonization from infection because they do not have data about the patient’s clinical condition or the results of other laboratory tests. The only criteria for qualifying samples as positive are described in the “Materials and Methods” section.

Additionally, many urine samples received by the laboratory were from patients already on antimicrobial therapy, which could have biased the results and excluded susceptible isolates or led to a negative culture result, as well as an over-representation of resistant isolates due to the administered therapy before the urine samples were collected. Antimicrobial stewardship activities, such as continuous medical staff education on current microbiological test guidelines, should reduce such events to a minimum.

## 5. Conclusions

On the basis of the above analyses, the best solution will be to separate the uncomplicated UTI and the complicated UTI in local guidelines and then recommend nitrofurantoin (furazidin) and amikacin, respectively, in empiric therapy. The clinician should decide based on the presented symptoms and then correct or continue the antimicrobial therapy after receiving the result of a microbiological urine test.

## Data Availability

The data that support the findings of this study are available from the corresponding author (F.B.) upon reasonable request.
